# New Cobalt-Mediated Radical Polymerization (CMRP) of Methyl Methacrylate Initiated by Two Single-Component Dinuclear β-Diketone Cobalt (II) Catalysts

**DOI:** 10.1371/journal.pone.0013629

**Published:** 2010-10-26

**Authors:** Feng Bao, Lingling Feng, Jie Gao, Zhifang Tan, Bin Xing, Rui Ma, Chunjie Yan

**Affiliations:** 1 College of Chemistry, Central China Normal University, Wuhan, China; 2 Department of Materials Science and Chemical Engineering, China University of Geosciences, Wuhan, China; University of Sydney, Australia

## Abstract

Two dinuclear cobalt complexes based on bis-diketonate ligands (ligand **1**: 3,3′-(1,3-phenylene)bis(1-phenylpropane-1,3-dione); ligand **2**: 3,3′-(1,4-phenylene)bis(1-phenylpropane-1,3-dione)) were successfully synthesized. The two neutral catalysts all showed satisfactory activities in the cobalt-mediated radical polymerization (CMRP) of methyl methacrylate (MMA) with the common initiator of azodiisobutyronitrile (AIBN). The resulting polymerizations have all of the characteristics of a living polymerization and displayed linear semilogarithmic kinetic plots, a linear correlation between the number-average molecular weight and the monomer conversion, and low polydispersities. Mono- or dicomponent low polydispersity polymers could be obtained by using the two dinuclear catalysts under proper reaction conditions. All these improvements facilitate the implementation of the acrylate CMRP and open the door to the scale-up of the syntheses and applications of the multicomponent low polydispersity polymers.

## Introduction

In recent years the development of the controlled/living techniques of radical polymerization (CRPs) was developed as an answer to the steadily increasing demand for new materials with controlled properties [1]–[4]. This concept is indeed a valuable strategy to provide a large range of polymers with well-defined molecular characteristics (length, composition and architecture), under non-very demanding conditions. The past few years have witnessed the rapid growth in the development and understanding of new CRP methods. All of these methods are based on establishing a rapid dynamic equilibration between a minute amount of growing free radicals and a large majority of the dormant species. Nowadays, nitroxide-mediated polymerization (NMP), atom transfer radical polymerization (ATRP), and radical addition fragmentation chain transfer (RAFT) are among the more popular CRP techniques [4]. NMP and ATRP rely on the same concept, which consists of decreasing the radical concentration in the medium and thus the probability of irreversible termination. This is achieved by reversible conversion of the growing macroradicals P· to dormant species PX. The persistent-radical effect (PRE) is the origin of the high propensity of the radicals to undergo reversible deactivation rather than self-coupling reactions. The lability of the cobalt–carbon bond under thermal and photolytic treatment, and thus the reversibility of its cleavage, makes cobalt complexes suitable candidates for PRE and regulation of CRP.

The first example of cobalt-mediated radical polymerization (CMRP), reported by Wayland et al., was the radical polymerization of acrylates in the presence of cobalt porphyrin complexes such as (tetramesitylporphyrinato)cobalt complex **1** [Co(TMP)] [5]. The cobalt complex reversibly end-caps the growing polymeric radicals under heating, which accounts for the equilibrium between dormant (P-Co^III^L) and active species (P·) (Scheme S2). Later, more efficient halogenated cobalt porphyrin complexes were designed, synthesized, and used to improve the polymerization kinetics. Moreover, the process was extended to alkylcobaloximes **2**, which are suitable photoinitiators for CMRP of acrylates [6]. One of the newest promising cobalt-mediated radical polymerization (CMRP) processes for conducting a successful controlled polymerization of VOAc is the recently reported [Co(acac)2]-mediated polymerization. This was initiated with 2,2′-azobis(4-methoxy-2,4-dimethyl-valeronitrile) (V-70) in the presence of bis(acetylacetonate)-cobalt(II) ([Co(acac)2]), resulting in the formation of poly-(vinyl acetate) (PVOAc) with predetermined Mn and low polydispersity. This system has been proposed to follow the organometallic radical polymerization mechanism. There is, however, an important feature of this efficient CMRP system that needs clarification [7]–[13]. The cobalt complexes used for ATRP had been reported by a few groups. One example was reported by Wang et al. using cobaltocene(II) as a catalyst, which, well known as a 19-electron compound, reportedly reduced the initiator and was oxidized from Co(II) to Co (III) to initiate the radical polymerization of MMA [14]–[16].

## Results and Discussion

Up to now, the catalyst activity and efficiency of cobalt catalyst are still not so high for CRPs (CMRP or ATRP). These facts mean that these catalysts coprecipitate with the polymer products after polymerization, coloring and contaminating the products. Thus, post-polymerization purification methods and in situ catalyst separations by liquid-liquid and solid-liquid (i.e., solid-supported catalysts) biphasic polymerization have been explored for CRPs to reduce the catalyst residue concentration in the polymers. However, these techniques lead to high costs and scale-up difficulties.

The most attractive approach to reduce the catalyst residue in CRPs products is to substantially increase the catalyst activity so that only a very small amount of catalyst is needed to catalyze the polymerization. Thus, no post-purification or catalyst recovery is necessary and the catalyst can be safely and economically left in the polymer products, as in polyolefins [17]. One approach to increase the catalytic activities is to introduce suitable ligands with multiple binding sites.

Herein we report the synthesis, reactivity, and polymerization behavior of two dinuclear cobalt catalyst (Scheme S1) capable of controlled radical polymerization of methyl methacrylate. In a comparison of bis(β-diketone) [ligand **1**: 3,3′-(1,3-phenylene)bis(1-phenylpropane-1,3-dione), ligand **2**: 3,3′-(1,4-phenylene)bis(1-phenylpropane-1,3-dione)] with β-diketone (acetylacetonate) ligands, the former have two binding sites and the latter have only one. Interestingly, the structures of the two bis(β-diketone) ligands could be regarded as a joint model of two acetylacetone ligands. Therefore, this communication could be considered as a continuous research to the former paper (Scheme S2).

The ligands and Co(II) complexes were synthesized by an improved procedure of the literature [18], as shown in Scheme S1. The synthetic way is easily performed to the previous report [19]. The single crystal of ligand **2** was grown in appropriate mixed solvents, and they were fit for X-ray crystallography description.

**Figure 1:**
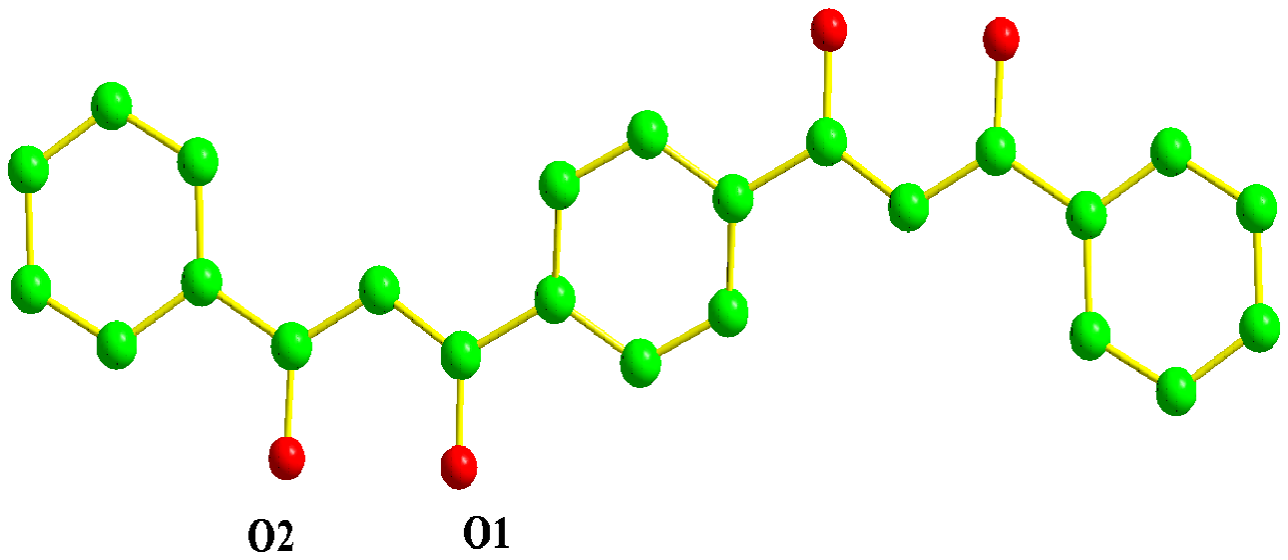
X-ray structure of ligand 5. Hydrogen atoms are omitted for clarity.

The structure of ligand **2** has already been reported by Soldatov et al [19]. Two different crystalline forms of the H2L2 were isolated in that paper. As the forms were distinct (as attested by solid state experimental characterization), they were thus confirmed as polymorphs. The crystal structure of **H2L2** (Figure **1**) shows a planar structure with the two diketonate units in trans- arrangement to maximized conjugation. The two crystal cell parameters of the ligand **2** obtained by Soldatov group are distinct to the ligand 2 reported by our group. Similarly, the ligand 2 of this paper could be seen as the third polymorphs.

Not long before, a cobalt complex with ligand **2** has already been reported and crystallographically characterized [19] as a trinuclear compound [Co_3_Py6L_3_]*4.11(CHCl_3_). Another recent paper reports the same compound as a water solvate and argues that it must be trinuclear, like other characterized members having the same stoichiometry [20]–[24].

### Cobalt-mediated radical polymerization of the dinuclear catalytic systems

Recently, R. Jérôme and his coworkers reported a system based on cobalt acetylacetonate [Co(acac)_2_] that imparts control to the radical polymerization of vinyl acetate initiated by 2,2′-azobis(4-methoxy-2,4-dimethyl valeronitrile), V-70, in the bulk at 30°C. The molar mass of poly(vinyl acetate) indeed changes linearly with monomer conversion, in good agreement with the predicted values. Moreover, the polydispersity is as low as 1.2 [7]–[13]. These observations are consistent with a mechanism based on reversible addition of the growing radicals to the cobalt complex and establishment of an equilibrium between alkylcobalt(III) and cobalt(II) complexes, that is, the dormant and the active species, respectively. A similar mechanism was previously proposed for acrylate polymerization mediated by cobalt porphyrin and cobaloxime complexes. However, the CMRP of vinyl acetate was initiated at higher temperature (60°C) by AIBN instead of V-70 in the presence of [Co(acac)_2_]. Under these conditions, the molar mass again increases with monomer conversion, the polymerization rate is higher, but the experimental molar masses are higher than predicted (M_n,theor_/M_n,SEC_ ≈0.5), which is evidence for higher probability of irreversible chain termination. Consistently, the molar mass distribution is much broader (Mw/Mn  = 2.0–3.5). Seemingly, the AIBN is not a preferable initiator for CMRP of MMA up to now. In this report, we make an attempt to use a common initiator instead of V-70 in the presence of CMRP. Under the using of the new bis(β-ketone) dinuclear cobalt complexes, the AIBN is an effective initiator in CMRP.

In addition, we also expected that the polymerization could be controlled. The results well coincided with our expection of obtaining higher activity in CMRP. Beyond our expectations, the molecular weight distributions of the obtained polymers showed an uncommon result. The polymerization results were reported in the following text.

**Figure 2:**
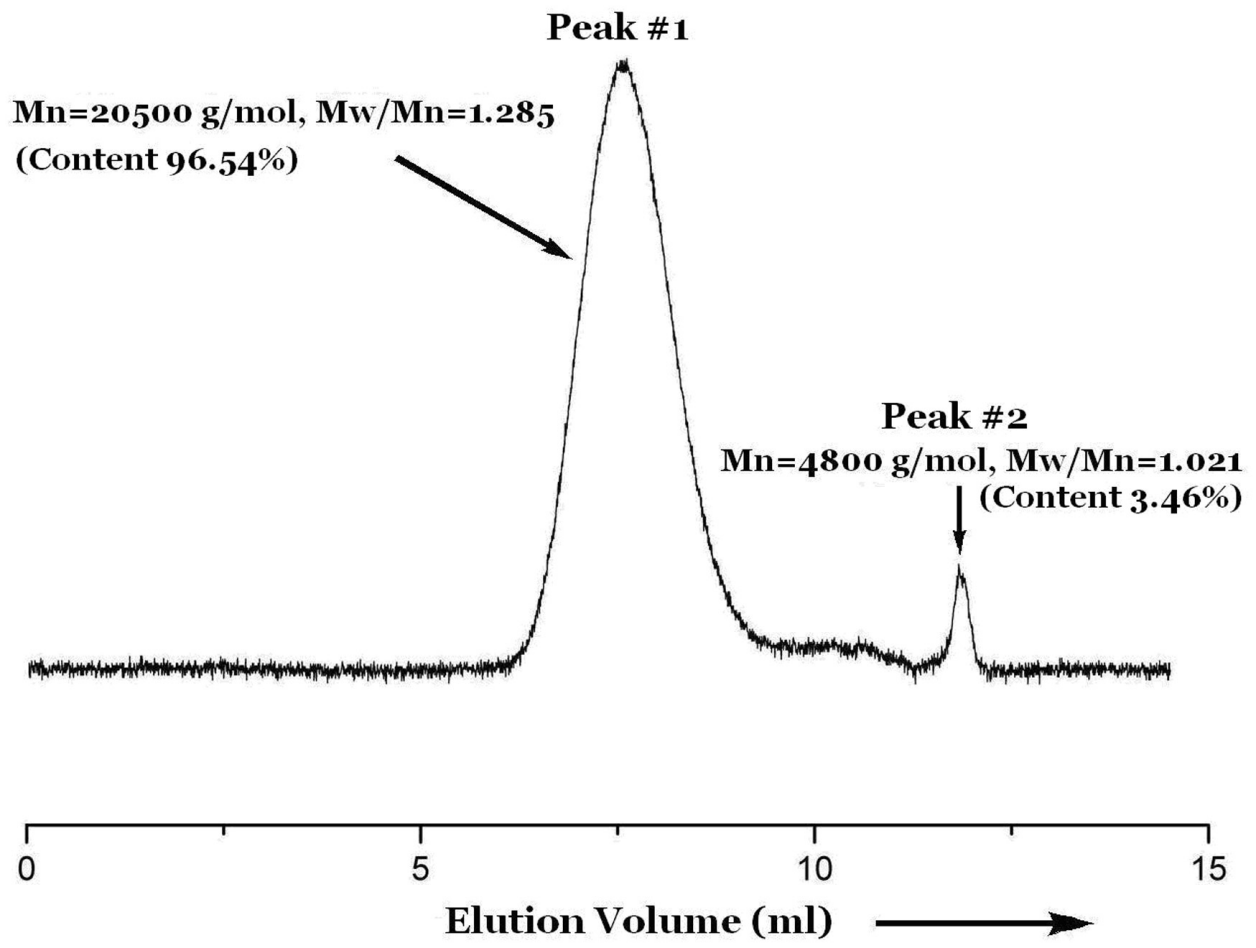
Size-exclusion chromatogram for Sample 6.

We carried out the polymerization of MMA catalyzed by **4**–**5** (Scheme S2) under conditions similar to those used with the cobalt catalyst reported by Matyjaszewski and Jérôme et al and the results were summarized in Table 1. When the polymerization of methyl methacrylate (MMA) is conducted in the presence of complex **5** under the same experimental conditions, clear polymer is formed for only one hour at 80°C (Table 1, sample **6**). More interestingly, the polymerization of MMA starts after this short period of time, and two independent controlled processes is observed by judging from the GPC curve of the polymer as portrayed in Figure **2**. The polymer shows two separate narrow distribution peaks in the GPC curve. The Mn of the polymer of peak #1 is 20500 g/mol and the Mw/Mn is 1.285. The content of the peak #1 in the total polymer is estimated to 96.54% by using Wyatt Technology (Astra 473) software. The Mn of the polymer of peak #2 is 8400 g/mol and the Mw/Mn is 1.021. Similar results are also found in many other polymerization products.

**Table 1:** Bulk cobalt mediated radical polymerization of methyl methacrylate in the presence of dinuclear complex **4** and complex **5**.

Complex	Sample number	[M]/[Co]	[Co]/[AIBN]	t [h]	Z^[b]^ [%]	Peak #1				Peak #2		
						M_n,sec_^[c]^ [gmol^−1^]	M_n,theor_^[d]^/M_n,sec_^[c]^	M_W_/M_n_	Content [%]	M_n,sec_^[c]^ [gmol^−1^]	M_w_/M_n_	Content [%]
**4**	**1**	320	3	4	66	16400	1.28	1.441	97.95	5950	1.008	2.05
**4**	**2**	320	3	8	92	25100	1.17	1.632	95.37	9420	1.032	4.63
**4**	**8**	320	2	6	65	17900	1.16	1.382	100	/	/	0
**4**	**3**	320	2	8	76	24400	1.00	1.566	100	/	/	0
**4**	**9**	320	2	2	39	12100	1.03	1.421	100	/	/	0
**4**	**10**	320	1	4	77	24300	1.02	1.273	100	/	/	0
**5**	**4**	600	3	6	78	34500	1.35	1.076	89.17	30700	1.001	10.83
**5**	**6**	600	2	1	29	20500	0.85	1.285	96.54	4800	1.021	3.46
**5**	**5**	600	2	2	54	23200	1.40	1.310	87.58	7820	1.015	12.42
**5**	**7**	600	2	4	66	29200	1.36	1.594	76.90	9470	1.007	23.10
**5**	**11**	600	2	5	73	33100	1.32	1.602	72.58	11890	1.010	27.42
**5**	**12**	600	1	4	55	30800	1.08	1.083	98.62	3100	1.025	1.38

[a] Bulk polymerization of MMA at 80°C. [b] The monomer conversion Z is determined gravimetrically after removal of the unconverted monomer in vacuo. [c] Determined by size-exclusion chromatography (SEC). [d]M_n,theor_ = ([M]_0_/[C_o_^II^]_0_)×M_mono_×Z.

As far as our knowledge goes, many dinuclear catalysts had been reported for using in ethylene or norbornene polymerization through coordination mechanism [25]–[27]. We are interested in comparing our results with those applications of dinuclear late transition metal complexes. In ethylene or norbornene polymerization, the results showed that the catalytic activity evidently increased and molecular weight distributions of the obtained polymers broadened. The observed effects can be explained on the basis of and steric and electronic effects and cooperative interactions of the two adjacent metal centers in dinuclear complexes. These two metal centers are electronically coupled through the ligand bridge. When one of the centers was activated at a given time, it had an electronwithdrawing effect on another center. The interaction between two metals made it possible to create more than one kind of active species during the polymerization. The two separate late metal complex units act as the mutual bulky steric effects for each other, which leads to the excellent catalytic performance.

Not long ago, a significant research about dinuclear catalyst system arouses our attention. Matyjaszewski group developed a highly active dinuclear copper-based catalyst of copper-based complex CuBr/N,N,N',N'-tetrakis(2-pyridylmethyl)- ethylenediamine (TPEN) for atom transfer radical polymerization [17]. The activator Cu^I^Br/TPEN existed in solution as dinuclear and mononuclear complexes in equilibrium but as a dinuclear complex in its single crystals. Therefore, both mononuclear and dinuclear complexes coexisted in the solution of CuBr/TPEN at a 1/1 molar ratio. There was fast exchange equilibrium between the dinuclear and mononuclear complexes. The facts of the real active species of CuBr/TPEN catalyst in solution might be the mononuclear species were found by the large differences in catalytic activity between CuBr/TPEN and CuBr/BPMPrA. Therefore, only single narrow molecular weight distribution peak was found in GPC curve.

Originally, we expected to get higher activity systems in CMRP by using the dinuclear cobalt complexes. It's a pleasant surprise to us, higher catalytic activities in CMRP is successfully obtained as expected. On the contrary to the ethylene polymerization, the molecular weight distributions of the obtained CMRP polymers did not broaden. Moreover, the polydispersity (PDI) of the two different constituents in Sample **6** (Table 1) are all very narrow (1.285 and 1.021). Similar to ethylene polymerization catalyzed by dinuclear catalysts, it has the same possibilities to create two kinds of active species during the CMRP for the electronwithdrawing effect on another center. As described in Scheme S3, a cobalt complex corresponds to polymerization equilibrium in CMRP of acrylates. In an individual polymerization process, the two kinds of active species catalyzed two independent cobalt-mediated radical polymerization processes. That is why we got the peculiar polymer of two distinct molecular weights as the GPC curve shown in Figure **2**. The very narrow polydispersities of the two polymer components indicate the two independent cobalt-mediated radical polymerization processes are controlled and living.

**Figure 3:**
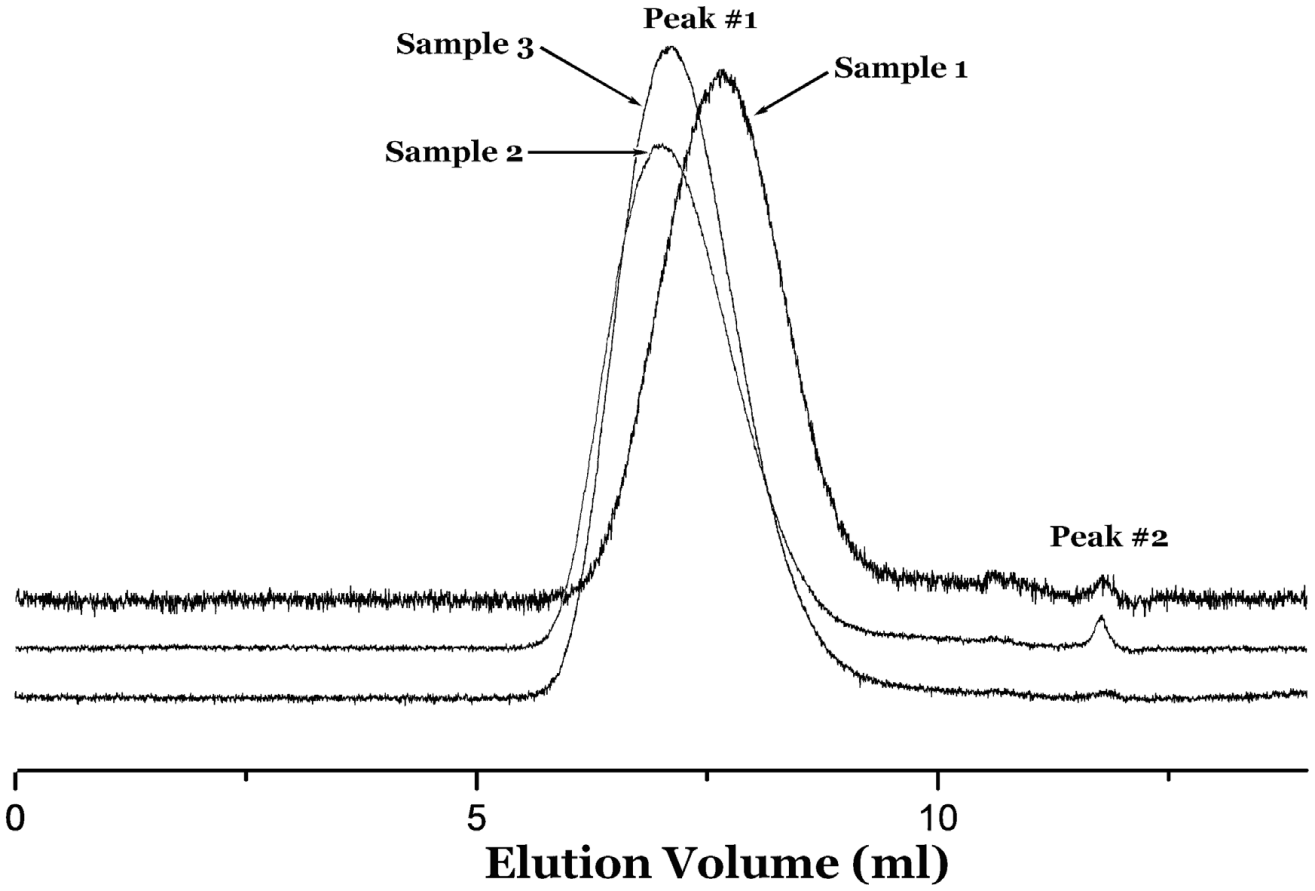
Size-exclusion chromatograms for Sample 1–3.

**Figure 4:**
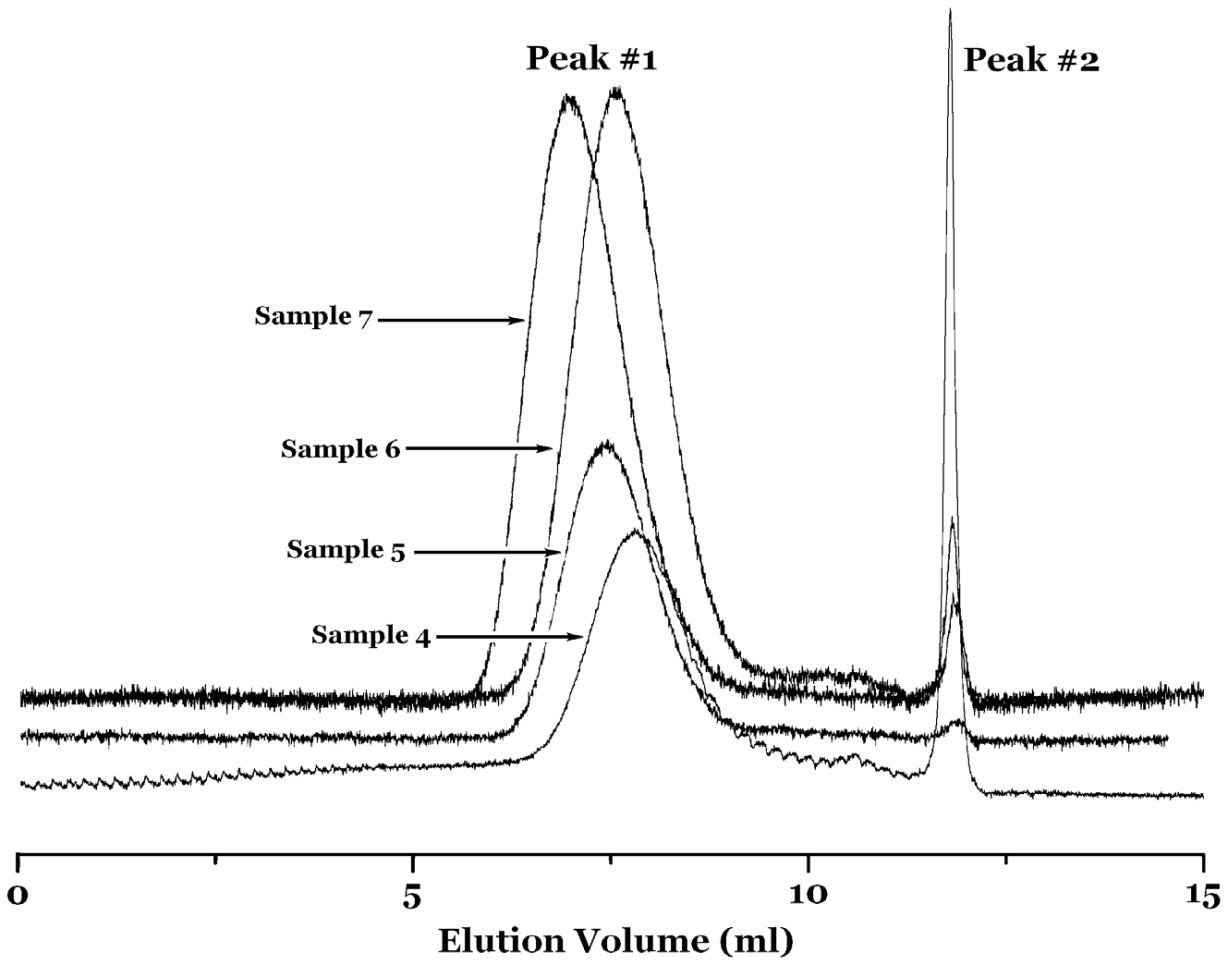
Size-exclusion chromatograms for Sample 4–7.

Other bulk CMRP results are summarized in Table 1 and the other GPC curves are shown in Figure **3** and Figure **4**. The results of two peaks in an individual GPC curves are found in most bulk CMRPs. Nonetheless, there still some polymers had only single narrow molecular weight distribution peak in GPC curve. It could be observed in Figure **3** (Sample **3**) and Table 1 (Sample **8**, **9**, **10**). From the above-mentioned GPC results, some unimodal distribution means only one active species worked in the CMRP with the catalyzation of a dinuclear cobalt complex under certain reaction conditions. Take all the GPC results shown in Table 1 into account, we could find the monocomponent polymer obtained by CMRP are all catalyzed by complex **4** (shown in Scheme S2) system. Similar results were not found in complex **5** systems. When will we get monocomponent narrow distribution polymers as well as when will we get two-component narrow distribution polymers, that are very significant questions to us. It is natural for us to explain these questions from the molecular structures of the two dinuclear complexes (complex 4 and complex 5). From Scheme S2, in complex 4, one benzoylacetone substituent group is in the para position to the other benzoylacetone substituent. Relatively, in complex 5, the benzoylacetone substituent group is in the ortho position to the other benzoylacetone substituent. These facts mean the two cobalt metal center in complex **5** is closer than in complex **4**. Therefore, for the higher electrophilic and steric effect, the complex **5** is more liable to generate two active species in CMRP. With a comparison to the bi-component polymers catalyzed by complex **4**, the mono-component polymers catalyzed by the same complex are all high molecular weight constituent.

**Figure 5:**
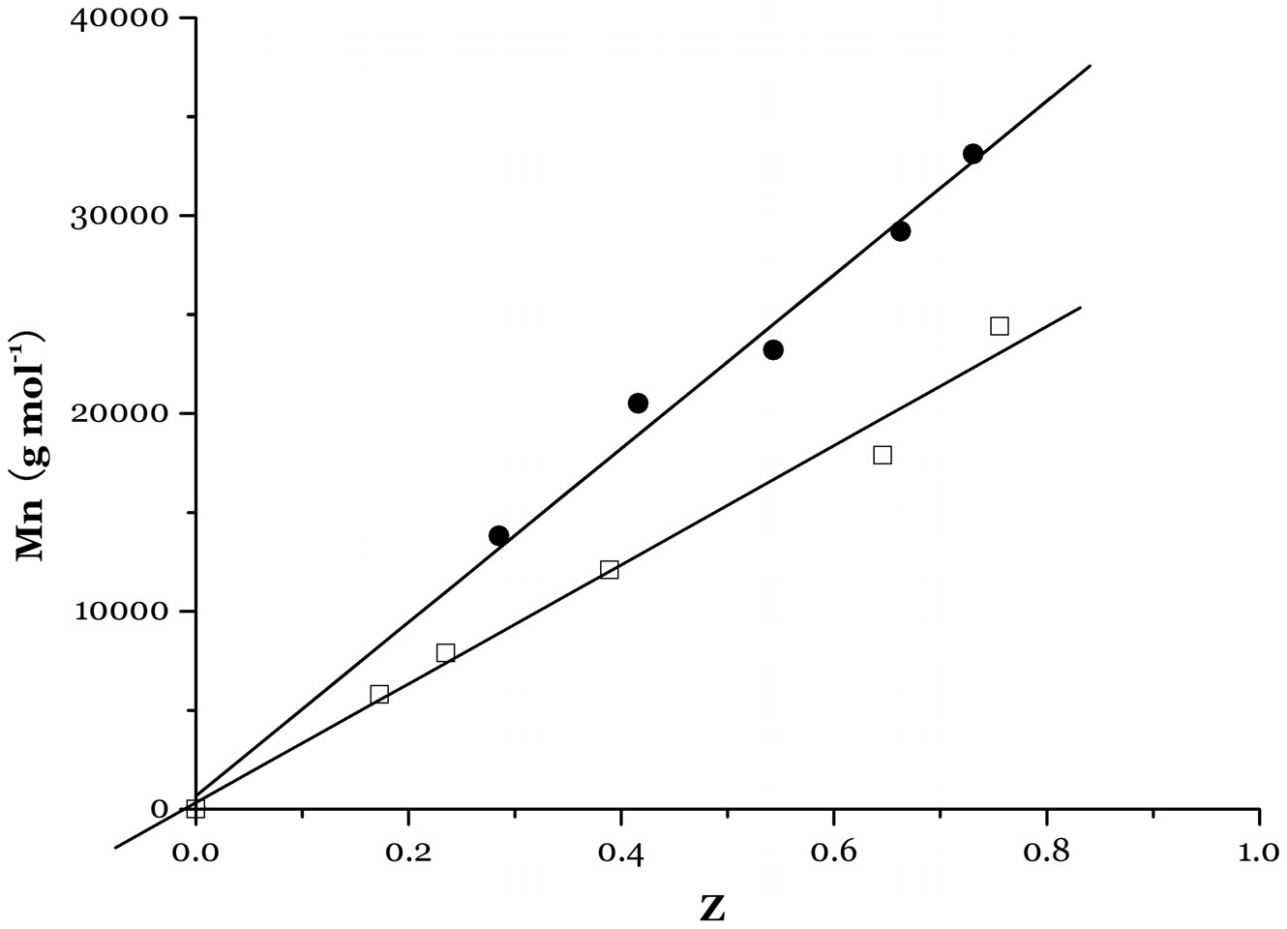
Dependence of PMMA molar mass Mn on monomer conversion Z for bulk polymerization of MMA at 80°C (for the primary constituent of the obtained PMMA). [MMA]/[Co(II)] = 600 (Table 1). (□ complex 4, • complex 5)

**Figure 6:**
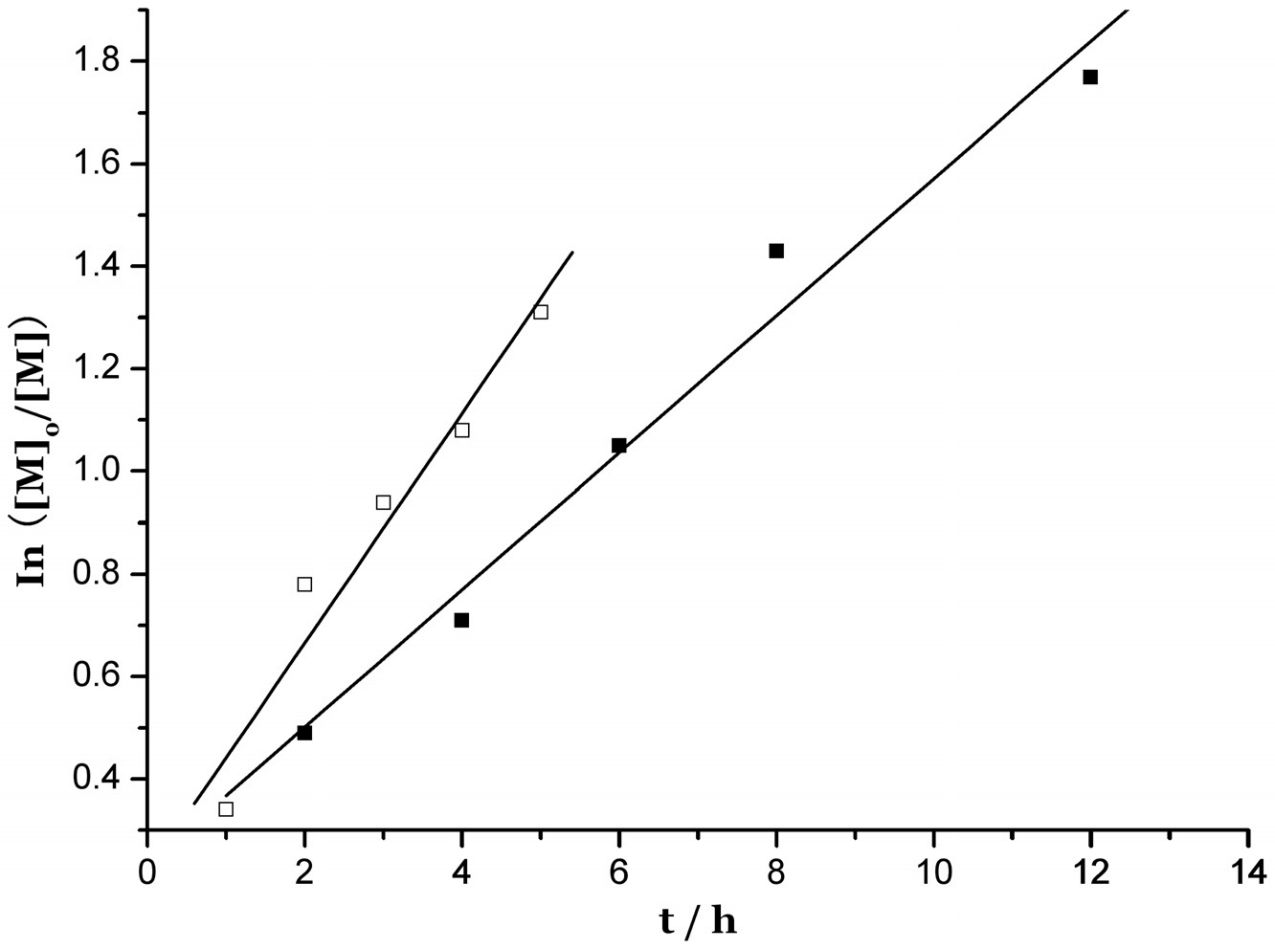
Time dependence of ln([M]_0_/[M]) for the bulk polymerization of MMA at 80°C (for the primary constituent of the obtained PMMA). [MMA]/[Co(II)] = 600 (Table 1). [M]_0_ and [M] are the MMA concentration at times 0 and t, respectively. (□ complex 5; ▪complex 4)

As expected, the molar mass of primary constituent of PMMA, is controlled by the [MMA]/[Co^2+^] ratio. Moreover, the polymerization of MMA starts after this period of time, and a controlled process is observed, as assessed by the linear increase of the molar mass with monomer conversion (Figure **5** and Figure **6**).

As a rule the polydispersity is very low (1.076≤Mw/Mn≤1.632) throughout the polymerization. The slight but significant increase in polydispersity with monomer conversion is observed in any controlled radical processes as result of irreversible termination reactions and, in this case, possible transfer reactions to monomer and/or polymer. Further evidence for the pronounced efficacy of this cobalt complex in mediating the radical polymerization of MMA can be found in the good agreement between experimental and theoretical molar masses (M_n,theor_/M_n,SEC_ = 1±0.40, Table 1, for the peak #1). The theoretical values were calculated under the assumption that one polymer chain is growing per cobalt complex unit, according to the mechanism proposed by Wayland and co-workers for the CMRP of acrylates (Scheme S3). ^1^H NMR and FT-IR analyses showed that the obtained polymers were more or less syndiotactic, similar to samples prepared with ordinary CRPs. There were almost no effects of AIBN on their tacticity. Lowering the temperature increased the racemic (rr) content, which would be expected for a free-radical polymerization. These also support the radical propagation mechanism with the cobalt catalysts.

The kinetics of this CMRP of MMA are first-order in monomer, as assessed by the linear dependence of ln([M]_0_/[M]) versus time (Figure **5** and **6**), which confirms that the macroradical concentration remains essentially constant and that irreversible chain termination reactions are quite restricted. In contrast to vinyl acetate polymerization reported by Jérôme et al, an induction period is not evidently observed in the radical polymerization of MMA, which would be almost simultaneous of the radicals generated in situ to convert Co^II^ into Co^III^―P with the polymerization beginning. During this beginning period of CMRP, a drastic change in the color of the medium, from purple to dark brown-green, is observed, consistent with the suggested change in oxidation state of the cobalt complex.

**Figure 7:**
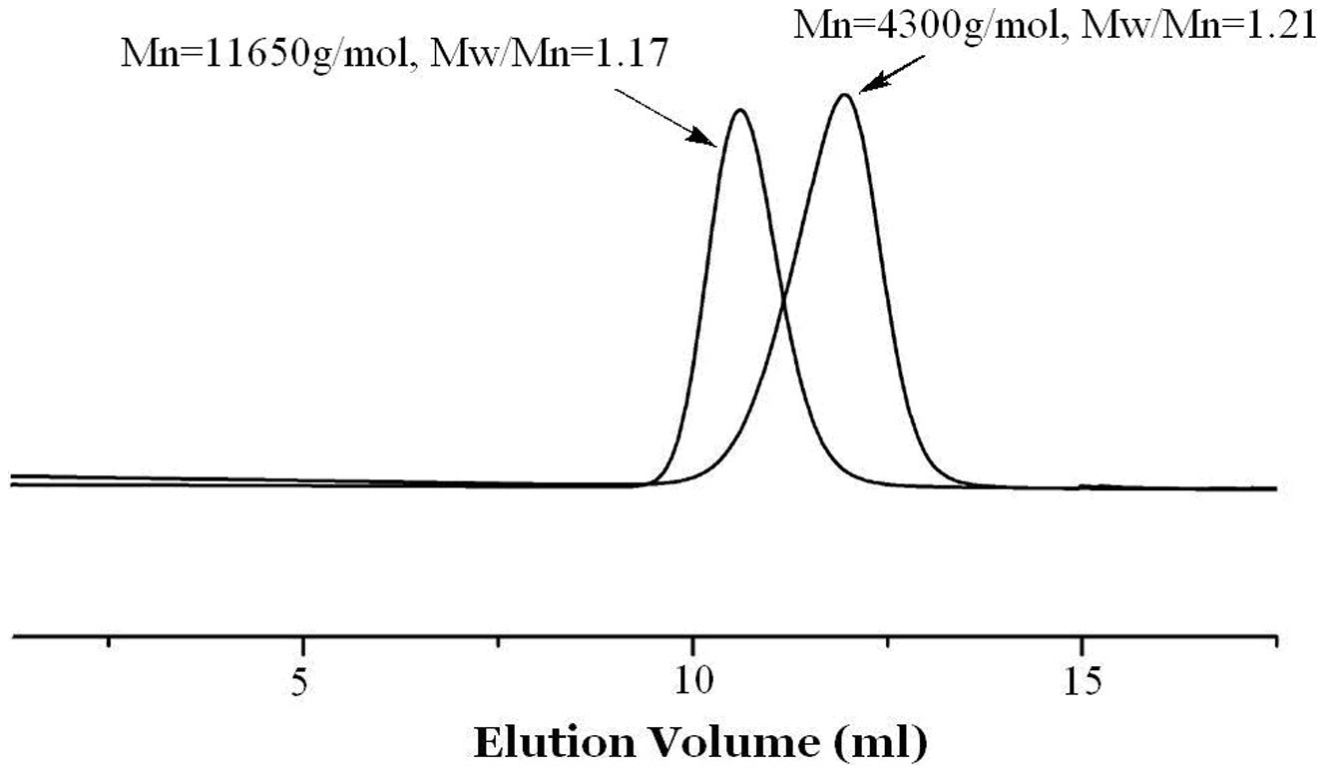
Size-exclusion chromatogram for the macroinitiator and block copolymer.

MMA oligomers (Mn = 4300 gmol^−1^, Mw/Mn = 1.21), preformed in bulk at 50°C with AIBN in the presence of complex **4**, were collected and purified by repeated precipitation in heptane, and finally used as macroinitiators for MMA polymerization. As shown in Figure **7**, resumption of the MMA polymerization was successful, as assessed by the shift of the SEC chromatograms with time towards lower elution volumes. A new MMA homopolymer (Mn = 11650 g/mol) was obtained with its maintained narrow polydispersity (PDI = 1.17), and a very small amount of unconverted macroinitiator. This observation paves the way for the synthesis of block copolymers by sequential polymerization of MMA and any comonomer whose polymerization is controlled by the same initiator/Co complex system. In that case, exchange of Co complexes is required between the consecutive polymerization steps.

### Conclusion

In conclusion, we have developed the first dinuclear catalyst for rapid, living MMA polymerization as demonstrated by kinetics studies and GPC data. The two unique, symmetrical, dinuclear cobalt complexes can be easily synthesized in one step from the bis-diketonate ligands. The neutral catalysts show satisfactory activities in MMA living polymerization. Interestingly, one or two low polydispersity polymers could be obtained by using the two dinuclear catalysts under proper reaction conditions. As we know, in precision gel permeation chromatographic (GPC) work, the calibration curve is obtained by using standard polystyrenes or other low polydispersity polymers. A general idea is to make “cocktails” – mixtures that contain several standards. That way, we don't have to make so many injections. But don't make the polymers too close. Therefore, the syntheses of multicomponent low polydispersity polymers have significant values in polymer chemistry. In this study, our successful producing two-component low polydispersity PMMA confirm the feasibility of multicomponent low polydispersity polymers syntheses. The further study of the multinuclear catalysts in CRP is currently under investigation and will be published shortly.

## Materials and Methods

All manipulations involving air- and moisture-sensitive compounds were carried out under an atmosphere of dried and purified nitrogen using standard Schlenk and vacuoline techniques. Solvents were purified using standard procedures. Reagents of the 2,2′-azobisisobutyronitrile (AR) were obtained from Aldrich and used without further purification. Methyl methacrylate were dried over CaH_2_, and then freshly distilled under vacuo prior to use. Molecular weights and molecular weight distributions were determined using gel permeation chromatography (GPC) coupled with multiangle laser-light scattering (MALLS). The system included a Styragel® HMW 6E GPC column (7.8×300 mm), a Wyatt OPTILAB RI detector, and a Wyatt multiangle laser-light scattering detector (DAWN E). The MALLS operated at 18 angles, from 26° to 149° and was equipped with a He-Ne laser (690 nm). The column and the RI detector were set at 40°C. The mobile phase was THF and the polymer solutions of injected sample were filtered through 0.2 µm syringe filters before injection. The molecular weights and polydispersity indexes were measured for the main peaks and were calculated using Wyatt Technology (Astra 473) software. The MALLS detector allowed the calculation of absolute molecular weights. Infrared spectra were recorded on polymer-KBr pellets with a Bruker EQUINOX55 FT-IR spectrophotometer in the region of 4000∼400 cm^−1^. NMR spectra were obtained using a Varian 400 MHz at room temperature in CDCl_3_ solution using tetramethylsilane as internal standard.

### Synthesis of two cobalt complexes

Complex **5**: The ligand **1** of 3,3′-(1,3-phenylene)bis(1-phenylpropane-1,3-dione) was synthesized according to Pikramenou and his coworkers' previous work.

To a DMF (5 ml) solution of 1,3-bis(3-phenyl-3-oxopropanoyl)benzene (40 mg, 0.11 mmol) were added triethylamine (0.31 ml, 0.22 mmol) and cobalt(II) chloride (16 mg, 0.12 mmol) dissolved in DMF (5 ml). The brown precipitate was collected for further characterized in about 80% yield. Elemental analysis calculated for C_48_H_32_Co_2_O_8_: C 67.46, H 3.77; found: C 67.52, H 3.68. However, attempts to get mono-crystal structure of complex **5** were unsuccessful. ^1^H NMR of Complex **5** (400M Hz, CDCl_3_): 8.59 (H, m), 7.50–7.59(12H, m), 6.94(6H, s). FT-IR(KBr): 2924, 1661, 1599, 1508, 1454, 1424, 1292, 1208, 1106, 1057, 744, 700 cm^−1^.

Complex **4**: The ligand **2** of 3,3′-(1,4-phenylene)bis(1-phenylpropane-1,3-dione) was synthesized improving to the synthesis of ligand **1**. Elemental analysis calculated for C_24_H_18_O_4_: C 77.82, H 4.90; found: C 77.90, H 5.01. The single-crystals (yellow crystals) of Ligand **2** were grown in ethanol, and the ORTEP plot of structure is shown in Fig. 1. (CCDC reference number is 678685.)

The complex **4** (as brown crystal) was obtained analogously to the preparation of complex **1**. Elemental analysis calculated for C_48_H_32_Co_2_O_8_: C 67.46, H 3.77; found: C 67.72, H 3.85. However, attempts to get mono-crystal structure of complex **4** were unsuccessful. ^1^H NMR of Complex **4** (400M Hz, CDCl_3_): 8.70 (6H, m), 7.57–7.64(12H, m), 6.99(6H, s). FT-IR(KBr): 2986, 1709, 1611, 1497, 1439, 1313, 1229, 1146, 1089, 1016, 750, 713 cm^−1^.

### General polymerization procedure

AIBN (98.4 mg, 0.60 mmol) and cobalt complex **4** (85.4 mg, 0.1 mmol) and solvent (xylene, 10 ml) were placed in a 50 mL flask and degassed by three vacuum/nitrogen cycles. Dry, degassed methyl methacrylate (11 mL, 120 mmol) was then added by syringe under nitrogen. The purple mixture was stirred after heated at 80°C. After a few hours, the color changed from purple to dark brown-green. Polymerization occurred for no less than 4 h, after which the viscosity of the solution increased substantially. After a given time, the mixture was diluted with THF and poured into 10-fold methanol. The resulting precipitated polymers were treated by filtering, washing with methanol several times, and drying under vacuum at 40°C to a constant weight. Some samples were preceded at different reaction times, and monomer conversion was determined by weighing the collected polymer after removal of the unconverted monomer in vacuo.

PMMA oligomers of only one constituent (460 mg, M_n,SEC_  = 4300 gmol^−1^, Mw/Mn  = 1.21) were prepared at 50°C with AIBN in the presence of complex **4** and collected at low monomer conversion by repeated precipitation in heptane. They were dissolved in degassed MMA (2.5 mL, 26.5 mmol) and heated at 50°C to resume the controlled radical polymerization.

## Supporting Information

Scheme S1 Syntheses of dinuclear cobalt(II) complexes
(0.42 MB TIF)

Scheme S2 Structure of cobalt(II) complexes (1–5)
(0.39 MB TIF)

Scheme S3 Equilibrium between dormant and active species in CMRP of acrylates
(0.06 MB TIF)
